# “Implementing a Multicomponent Sensory Support Intervention in Long‐Term Care: Staff Perspectives from the SENSE‐Cog Residential Care Feasibility Study”

**DOI:** 10.1002/alz70858_104901

**Published:** 2025-12-26

**Authors:** Iracema Leroi, Petya Grigorova, JP Connelly

**Affiliations:** ^1^ School of Medicine and Global Brain Health Institute, Trinity College Dublin, Dublin, Ireland; ^2^ Dementia Trials Ireland, Dublin, Ireland

## Abstract

**Background:**

Dementia affects approximately 64,000 people in Ireland, with 20,000 residing in long‐term care (LTC) facilities. Sensory impairments, including hearing and vision issues, are highly prevalent among residents with dementia (RwD) in LTC, affecting 75–90% and 40% of this population, respectively. These impairments often go undetected and unmanaged, exacerbating cognitive decline, social isolation, agitation, and reduced quality of life. The SENSE‐Cog Residential Care (SENSE‐Cog RC) feasibility study aimed to address this gap by evaluating a multicomponent sensory support intervention (SSI‐RC) designed to improve sensory health for RwD in LTC settings. The SSI‐RC intervention comprised four levels: sensory assessments and aids (glasses, hearing aids, listening devices) for residents, staff training (including “Sensory Champions”), environmental modifications, and organisational‐level mapping of sensory care pathways.

**Method:**

This study explored the experiences of LTC staff involved in the intervention arm of the SENSE‐Cog RC feasibility trial. Semi‐structured interviews were conducted with 13 staff members, including Sensory Champions and LTC facility managers/Directors of Nursing, across four intervention‐arm facilities. Data were analysed thematically to identify key facilitators and barriers to implementation.

**Result:**

Findings indicated that the intervention was largely integrated into existing routines without significant disruption. However, challenges emerged across the four intervention levels. At the resident level, some RwD struggled to adapt to sensory aids, while others perceived no need for them. At the staff level, time constraints made it difficult to maintain sensory devices, complete personal sensory plans, and follow up on adherence. Sensory Champions highlighted the need for strong managerial support and teamwork to ensure RwD benefited from their sensory aids. At the environmental level, maintaining optimal lighting and noise levels required ongoing effort, adding to staff responsibilities. At the organisational level, collaboration with external providers needed further streamlining to ensure consistent and effective sensory care pathways.

**Conclusion:**

These findings highlight the importance of a structured, holistic approach to sensory care that aligns with the needs of RwD, staff, and the LTC environment. Insights from this study will guide further refinement of the intervention, enhancing its feasibility and scalability to improve quality of life for RwD and support LTC staff.

